# Population Physiologically Based Pharmacokinetic Modeling for the Human Lactational Transfer of PCB-153 with Consideration of Worldwide Human Biomonitoring Results

**DOI:** 10.1289/ehp.11519

**Published:** 2008-07-24

**Authors:** Laurel E. Redding, Michael D. Sohn, Thomas E. McKone, Jein-Wen Chen, Shu-Li Wang, Dennis P. H. Hsieh, Raymond S.H. Yang

**Affiliations:** 1 Division of Environmental Health and Occupational Medicine, National Health Research Institutes, Zhunan, Miaoli, Taiwan; 2 Lawrence Berkeley National Laboratory, Berkeley, California, USA; 3 University of California, Berkeley, California, USA; 4 Graduate Institute of Occupational and Industrial Health, Kaohsiung Medical University, Kaohsiung, Taiwan; 5 Quantitative and Computational Toxicology Group, Department of Environmental and Radiological Health Sciences, Colorado State University, Fort Collins, Colorado, USA

**Keywords:** body burden, exposure reconstruction, human milk biomonitoring, lactational transfer, PCB-153, physiologically based pharmacokinetic modeling, polychlorinated biphenyls, reverse dosimetry

## Abstract

**Background:**

One of the most serious human health concerns related to environmental contamination with polychlorinated biphenyls (PCBs) is the presence of these chemicals in breast milk.

**Objectives:**

We developed a physiologically based pharmacokinetic model of PCB-153 in women, and predict its transfer via lactation to infants. The model is the first human, population-scale lactational model for PCB-153. Data in the literature provided estimates for model development and for performance assessment.

**Methods:**

We used physiologic parameters from a cohort in Taiwan and reference values given in the literature to estimate partition coefficients based on chemical structure and the lipid content in various body tissues. Using exposure data from Japan, we predicted acquired body burden of PCB-153 at an average childbearing age of 25 years and compared predictions to measurements from studies in multiple countries. We attempted one example of reverse dosimetry modeling using our PBPK model for possible exposure scenarios in Canadian Inuits, the population with the highest breast milk PCB-153 level in the world.

**Results:**

Forward-model predictions agree well with human biomonitoring measurements, as represented by summary statistics and uncertainty estimates.

**Conclusion:**

The model successfully describes the range of possible PCB-153 dispositions in maternal milk, suggesting a promising option for back-estimating doses for various populations.

Before the 1970s, polychlorinated biphenyls (PCBs) had been used rather extensively in industries involving the manufacture of transformers, capacitors, and noncarbon copying papers. Despite the subsequent banning of PCBs because of their chemical stability and lipophilicity, PCBs continued to be an environmental and human health concern through bioaccumulation and biomagnification. Because humans are at the top of the food chain, it is not surprising that PCBs are consistently found in a variety of human tissues.

One of the most serious human health concerns from environmental contamination of PCBs is their presence in breast milk. Indeed, PCBs have been detected in milk samples from lactating mothers in the United States (Greizerstein et al. 1999; Schecter et al. 1998), Japan (Inoue et al. 2006; Suzuki et al. 2005), Spain (Angulo et al. 1999; Ramos et al. 1997), Taiwan (Wang SL, Chen JW, unpublished data), and all over the world (Dewailly et al. 1996; Glynn et al. 2001; Polder et al. 2003; Vartiainen et al. 1997). This is a serious human health concern because milk, with its high lipid content, represents a concentrated delivery mechanism of PCBs to infants. Furthermore, a number of human epidemiologic and animal experimental studies have established an association between neuro-developmental and neurobehavioral deficits and PCB exposure (Huisman et al. 1995; Jacobson et al. 1990; Tilson et al. 1990). At the cellular and molecular levels, exposure to PCBs during the developmental stage is known to disrupt thyroid hormone homeo-stasis and dopamine levels in the brain (Goldey et al. 1995; Seegal et al. 1997).

Given the human biomonitoring levels in breast milk worldwide, how can we effectively use such information? In this article, we present an approach to render such human biomonitoring results useful by using physiologically based pharmacokinetic (PBPK) modeling. We first transformed an earlier PBPK model for lactational transfer of PCB-153 in mice (Lee et al. 2007) into a PBPK model for a nonpregnant human female at an average childbearing age of 25 years. We focused on PCB-153 because it is the most prevalent PCB congener detected in human tissue, often representing around 27–30% of the total detected PCB congeners in human tissues (Inoue et al. 2006; Kiviranta et al. 2005). We then predicted the body burden buildup of PCB-153 from birth over a 25-year period based on realistic exposure levels found in foods, as reported for the Japanese population (Akutsu et al. 2005). In doing so, we incorporated all age-related physiologic changes during the first 25-year life span of a human female. Next, we transformed the PBPK model to a lactating 25-year-old woman by incorporating all the physiologic changes related to pregnancy and childbirth. Using this model, we predicted milk levels of PCB-153 using three sets of values (minimal, median, maximal) for the most sensitive parameters based on actual data reported in the literature. We found model predictions of PCB-153 in breast milk to bracket the human biomonitoring data found worldwide. We addressed uncertainties and variability through the modeling but did not conduct parameter estimation. Using this PBPK model, we carried out reverse dosimetry simulation to suggest possible exposure scenarios leading to the breast milk concentrations of PCB-153 in Canadian Inuits being the highest in the world.

## Materials and Methods

### PBPK model development

**Figure 1:**
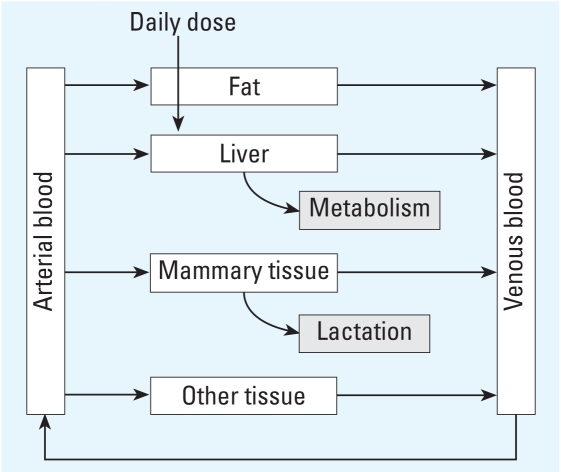
Five-compartment model of PCB-153 transfer during lactation.

We developed a PBPK model to predict the concentration of PCB-153 in human milk. Our model was derived from a model for PCB-153 transfer in pregnant and lactating mice (Lee et al. 2007). We limited the complexity of the human PBPK model to a five-compartment model consisting of four well-mixed tissue groups—liver, fat, mammary tissue, and rest of the body—and a mixed blood compartment (Figure 1), because available human data did not justify a more refined model, nor was one needed for our population-scale, multiyear model assessments/predictions.

All tissues in the model are flow-limited. PCB-153 is input directly into the liver, where metabolism occurs with a first-order metabolic coefficient allometrically extrapolated from the mouse value reported by Lee et al. (2007). We used postdelivery body weight measured at Taizhong hospital in Taiwan; this study also involved determining PCB concentration in milk, cord blood, and maternal venous blood. We modeled postpartum weight loss via formulas from Haiek et al. (2001). We used physiologic parameters for nursing women from Gentry et al. (2003), Fisher et al. (1997), and Byczkowski and Fisher (1995) (Tables 1 and 2).

Akutsu et al. (2005) reported daily intakes of PCB-153 in Japan of 0.00125–0.13 μg/kg/hr (median, 0.0068 μg/kg/hr). De Amici et al. (2001) and Fisher et al. (1997) reported milk production rates between 0.0033 and 0.06 L/hr (median, 0.0317 L/hr). We simulated population-scale exposure by uniformly sampling from this range of intakes and milk production rates.

To facilitate the data and statistical analyses, we coded our final model using R statistical software, version 2.7.2 (R Foundation for Statistical Computing 2008), an integrated suite of software facilities for data manipulation, calculation, and graphical display.

### Calculation of partition coefficients

We calculated partition coefficients (Table 1) using methods from Parham et al. (1997), who described calculations to determine the adipose:plasma and adipose:blood coefficients for any PCB using the structural properties of that PCB. Coefficients for other tissues were determined by multiplying the adipose:blood coefficient by an adjustment factor related to the lipid composition of the target tissue. We used adjustment factors, defined as


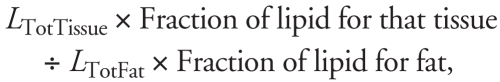


where *L*_Tot_ = fraction of neutral lipids + 0.3 × fraction of nonneutral lipids in a tissue, as described by Parham et al. (1997), or calculated from DeJongh et al. (1997). We examined the lipid fractions of tissues from a number of sources in the literature, and they were very similar. We do not believe that the minor differences of those published results would significantly influence the outcome of our PBPK modeling. Thus, we chose to use the values given by Parham et al. (1997) and DeJongh et al. (1997). The partition coefficient for the body compartment was the average of the partition coefficients for brain, skin, and muscle. We obtained the distribution of lipids of mammary tissue from Sakai et al. (1992). We then calculated the adjustment factor for mammary tissue.

### Model simulations to build up body burden through different developmental stages and to incorporate physiologic changes of lactating women

We simulated individuals beginning at age 0. Body weight, blood volume, fat volume, and cardiac output were evaluated according to developmental stages by age group (0–1 year, 1–5 years, 5–10 years, 10–15 years, and > 15 years) using values reported by Haddad et al. (2001) and Price et al. (2003). We assigned mammary tissue volume a very low estimated value for ages < 13 years (an average value for the onset of puberty) and its final lactational value at ages > 13 years. We assumed an average childbearing age of 25 years, and thus, in the simulations, lactation is assumed to begin at this age.

**Figure 2:**
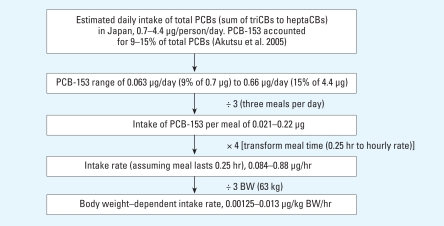
Derivation of input exposure dose for PBPK modeling of loading body burden of PCB-153 in a 25-year-old woman. BW, body weight.

The exposure input derived for PBPK modeling of a 25-year-old woman throughout her life is shown in Figure 2. Akutsu et al. (2005) reported that the exposure of Japanese to PCBs was in the range of 0.7–4.4 μg/person/day; the dominant congener found was PCB-153, which accounted for 9–15% of total PCBs. Thus, we derived an estimation of 0.063–0.66 μg/person/day exposure of PCB-153 in humans, as shown in Figure 2. We assumed further that this daily dose is divided evenly among three meals and that each meal takes 15 min (0.25 hr) to consume. Taking into consideration the average body weight of a 25-year-old woman to be 63 kg, we finally derived the bodyweight–dependent intake rate of PCB-153 to be 0.00125–0.013 μg/kg body weight/hr (Figure 2).

**Figure 3:**
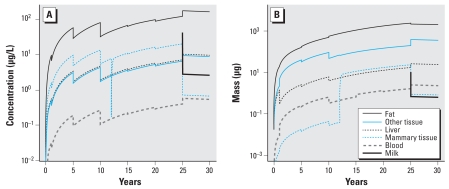
PCB-153 body-burden predictions for 1 of the 1,000 model simulations. In this model, we assumed that mammary tissue develops at 13 years of age and that lactation begins at age 25.

**Figure 4:**
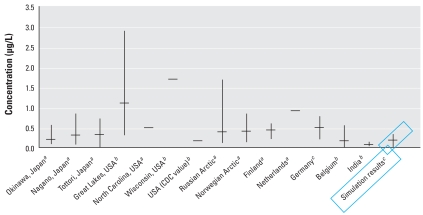
Mean and range of concentrations of PCB-153 in plasma (*^a^*), serum (*^b^*), and whole blood (*^c^*) of populations from worldwide geographic locations. Data from Covaci et al. (2002) for Belgium and Rusiecki et al. (2005) for India; all other data from Minh et al. (2006).

**Figure 5:**
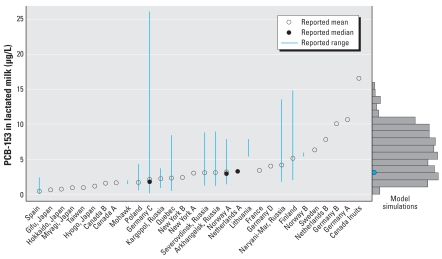
Histogram of model simulations (right) compared with global measurements of PCB-153 in breast milk (left). The uncertainty in the model predictions results from uncertainty in the daily PCB-153 intake and in milk lactation rate. The blue circle in the model simulation bar chart indicates the mean prediction. Data from Abraham et al. (1998), Germany; Angulo et al. (1999), Spain; Dewailly et al. (1994), Canadian Inuits; Glynn et al. (2001), Norway; Greizerstein et al. (1999), Canada A and B, Mohawk, Quebec, New York A and B, Netherlands A and B, Lithuania, France, Norway B, Germany A and B; Inoue et al. (2006), Japan; Jaraczewska et al. (2006), Poland; Patterson et al. (1994), Sweden; Polder et al. (2003), Russia; Vartiainen et al. (1997), Finland; and Wittsiepe et al. (2007), Germany C.

We addressed uncertainties and variability in our PBPK model for lactational transfer of PCB-153 in women by propagating such uncertainties and variability in the inputs in the model using Latin Hypercube sampling; this eventually led to a much more robust probability distribution for the population PBPK modeling of milk contents of PCB-153 in lactating women. In the model, we performed no parameter estimation to data comparisons shown in Figures 3–5. Our focus in this work was to observe and comment on the fidelity of a population-scale forward model derived from independent sources of information compared with worldwide measurements of PCB-153.

## Results

### PBPK model simulations of PCB-153 contents in serum, plasma, whole blood, and milk compared with worldwide human bio-monitoring data

Blood and tissue concentrations for a 25-year-old woman generated by this model were within ranges found in the literature. Initial sensitivity analyses indicated that milk production rate and initial fat content of PCB-153 were sensitive parameters for milk concentration of PCB-153. When we used the minimal, median, and maximal values of these sensitive parameters for model simulation, as well as daily intake values reported in the literature, the resulting three curves encompassed the human milk monitoring data published by various investigators (Angulo et al. 1999; Dewailly et al. 1996; Glynn et al. 2001; Greizerstein et al. 1999; Inoue et al. 2006; Polder et al. 2003; Ramos et al. 1997; Schecter et al. 1998; Suzuki et al. 2005; Vartiainen et al. 1997). The final simulation results for PCB-153 concentration in milk over a lengthy period of exposure were indistinguishable if the exposure mode was assumed to be via three meals daily or via a constant infusion, as long as the daily input was the same. Figure 3 shows an example of 1 of the 1,000 individuals simulated. The apparent jaggedness in the curves resulted from our rescaling of body parameters by body weight at the above-mentioned times and when mammary tissue develops. Figure 4 shows adult blood PCB-153 concentrations in various geographic locations in the world compared with simulation values.

Figure 5 shows a histogram of PCB-153 predicted in breast milk compared with global measurements reported in the literature. The range and spread in the model simulations, caused by uncertainty only in intake and lactation rate, span the range in the measurements. The mean model prediction also appears to be quite close to many of the means reported in the literature.

### Reverse dosimetry modeling

**Figure 6:**
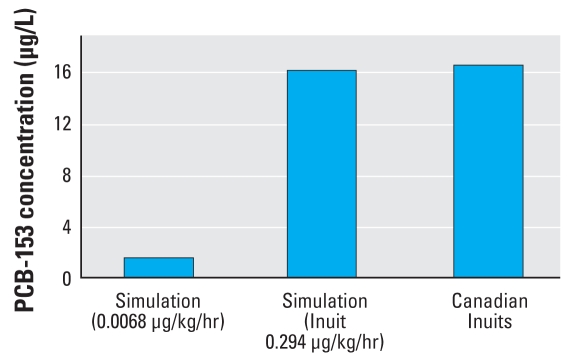
Breast milk PCB-153 concentrations from Canadian Inuit populations compared with the simulated PCB-153 milk concentration generated using the median oral intake dose of 0.0068 μg/kg/hr from Akutsu et al. (2005) and the simulated estimated Inuit dose of 0.294 μg/kg/hr.

Another application of a PBPK model is to reconstruct a possible exposure scenario from a given tissue level of PCB-153. By varying or sliding the intake dose, or other relevant physiologic parameters, we can obtain the PCB-153 milk concentration of interest. For example, Dewailly et al. (1994) found a group of Canadian Inuits to have a particularly high level of milk PCB-153 (16.59 μg/L). In fact, the milk content of PCB-153 among these Canadian Inuits is among the highest in the world (Figure 5). Similarly, the serum PCB-153 concentration of a group of fishermen from the same region is 2,457 ng/g lipid (Dewailly et al. 1994), or 14.7 μg/L in blood if we assume that blood is 0.6% lipid (Sakai et al. 1992). We raised the oral intake dose of PCB-153 of the model until we obtained mean milk levels of PCB-153 close to those reported by Dewailly et al. (1994) (Figure 6). More sophisticated approaches to exposure reconstruction are available (see, for example, Allen et al. 2007; Sohn et al. 2004) but were not needed for this work and are beyond the scope of this article. We were able to postulate an estimate of the daily intake dose of PCB-153 in this particular population: an intake rate of 0.294 μg/hr/kg body weight yielded a mean PCB-153 milk concentration of 16.18 μg/L. Because all other factors were held constant, the postulated dose is a rough estimate. However, this intake rate generates milk levels similar to those reported for populations in this region of Canada (Dewailly et al. 1994).

## Discussion

Based on actual human exposure data and parameter values reported in the literature, our PBPK model generates a range of results that encompasses human biomonitoring data of milk content of PCB-153 from all over the world. Therefore, the model has good predictive capability. Human biomonitoring data are increasingly being collected in the United States, Canada, and other countries in large-scale field studies. These studies are modeled after the efforts of the U.S. Centers for Disease Control and Prevention (CDC), which released its *Third National Report on Human Exposure to Environmental Chemicals* in the summer of 2005 (CDC 2005). This report, similar to its two predecessors but with expanded effort, contains biomonitoring data for the U.S. population for 148 environmental chemicals, grouped into 14 classes, over the period 2001–2002. Because so many chemicals are detected in the human body at very low levels, it is interesting to examine the health significance of these chemicals and ask what we can do about these data. The application of PBPK modeling and reverse dosimetry modeling in the present study may offer a glimpse of the utility of human biomonitoring data collected by the CDC and others.

In this model, we incorporated parameters and exposure scenarios that were as realistic as possible. Although we simplified our model from the mouse model (Lee et al. 2007), we maintained the most relevant compartments (fat, mammary tissue for lactation, liver for metabolism). We allometrically scaled the first-order rate constant for PCB-153 metabolism from the mouse value given by Lee et al. (2007) because we found no literature values for PCB-153 metabolism in humans. We divided the oral dose of PCB-153, given as a single daily dose in the literature (Akutsu et al. 2005), into three meals to more accurately represent intake. We have done simulations with an exposure scenario of constant infusion of the same daily dose over 24 hr, and the results were not different from the scenario of three meals per day.

We used appropriate age-dependent physiologic values (body weight, blood volume, fat volume, mammary tissue volume, and cardiac output) to simulate the period during which body burden of PCB-153 was acquired. Time intervals of 5 years were chosen as small enough to convey the changes brought on by growth but large enough to obtain literature-based values. The exception was mammary tissue volume and growth, for which no accurate values could be found in the literature. We assigned mammary tissue volume pre- and postpuberty values, with postpuberty values given those of a lactating woman. This parameter is probably subject to a certain inaccuracy because it is unlikely that upon puberty women acquire a mammary tissue volume equal to that observed during lactation. We did not model pregnancy separately.

In this model, we considered lactation to be a uniform phenomenon for simplicity. Even though the literature suggests that milk production and content vary throughout the day, as well as throughout lactation (Mitoulas et al. 2002), for the purposes of a PBPK model we assumed that lactational performance was constant, even over a wide range of maternal states (Butte et al. 1984). Most PBPK lactational models make similar assumptions with regard to the modeling of lactation (Fisher et al. 1997; Gentry et al. 2003; Lee et al. 2007).

The issue of the impact of body burden versus oral intake on the concentration of PCB-153 in the milk requires some discussion. Because PCB-153 is a highly lipophilic compound that is sequestered in adipose tissues, the distribution of lipids into milk is a function of specific lipid composition of the milk. Lipids in milk can come from three sources: *a*) directly from dietary intake (tri-acylglycerols originally derived from intestinal absorption are released from chylomicrons); *b*) from maternal adipose stores; and *c*) from synthesis by the mammary gland. Dietary status of the mother influences the source of fatty acids. If a mother is in a negative energy balance, lipids come mostly from adipose stores. If the maternal diet is low in fat but provides adequate energy through carbohydrates, a greater proportion of fatty acids is synthesized by the mammary gland. If maternal diet is high in fat, milk fatty acids will mostly originate from dietary intake (Del Prado et al. 1999). In the case of a negative energy balance, because adipose stores are mostly mobilized, acquired body burden of PCB-153 will be the most important source of PCB-153 in milk. In the case of a high-fat diet, because lipids mostly originate from diet, dietary intake of PCB-153 will be the principal source of PCB-153 in milk.

Lactation is metabolically demanding on the mother, and in most cases, maternal adipose stores must be mobilized to provide enough energy to the newborn. Studies in rats show that adipose stores are completely depleted at the end of a lactation cycle (Del Prado et al. 1999). Therefore, as lipid and thus PCB-153 body burdens are depleted, dietary PCB-153 constitutes a greater proportion of chemical found in milk. However, the appearance of dietary PCB-153 in milk is probably delayed: Brambilla et al. (2008) showed that cows fed a diet contaminated with polychlorinated dibenzo-*p*-dioxins (PCDDs) and polychlorinated dibenzofurans (PCDFs) produced contaminated milk 4 weeks later, whereas contamination tapered off within 75 days after supplement withdrawal. It is probable that a meal particularly high in PCB-153 will not directly result in higher levels in the milk.

**Figure 7:**
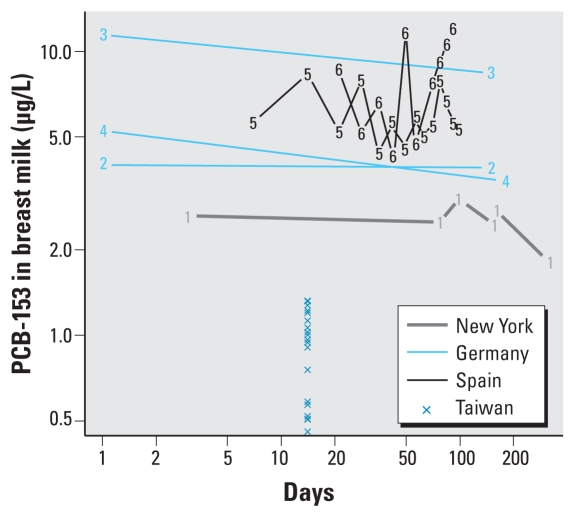
Time courses of PCB-153 concentrations in milk from lactating mothers from various countries. New York State data are from Schecter et al. (1998); each number 1 indicates the milk concentration value and time of sampling for six lactating women. Data for Germany are from Abraham et al. (1998), and the numbers 2, 3, and 4 represent time course data from three lactating women. Data for Spain are from Ramos et al. (1997), and numbers 5 and 6 indicate time course data for two lactating women. Xs represent single data points for 20 lactating women in Taizhong hospital in Taiwan.

We used a number of data sets for validation of this model (Figure 7): a pseudo-time course from different mothers of one geographical location [New York (Schecter et al. 1998)] and Taizhong hospital data; and actual time courses from individual mothers [Germany (Abraham et al. 1998); Spain (Ramos et al. 1997); New York (Schecter et al. 1998)]. Validation data were useful in verifying ranges of values but not necessarily in identifying trends of PCB-153 concentrations in milk. There is no clear trend in either the individual time courses or the population-based pseudo-time courses. This is not wholly unexpected in a population-based study because values are usually mean values and because such a cohort is subject to great inter-individual and intraindividual differences. Similarly, mean values and ranges of PCB-153 milk concentrations in different areas of the world are of limited use because diet and physiologic attributes differ throughout these areas and because we have no control over sample collection times and methods. However, they are able to validate the ranges and mean values of our simulation.

The agreement between model predictions and data in Figure 5 helps to support the level of complexity employed in this PBPK model. We condensed the Lee et al. (2007) PBPK model for PCB-153 in mice because sufficient data to parameterize an equivalent human model were unavailable. We also felt that they were not needed to make predictions at the global scale. Uncertainties in intake and lactation rates alone may cause model predictions as wide as or wider than the range of concentrations reported in the literature. This suggests that the limiting factor in improving the fidelity of the PBPK model lies more on understanding the inputs of the existing model (e.g., intake, lactation) than in increasing the complexity of the model by adding tissue compartments.

Although this model is useful in its ability to describe the distribution, absorption, metabolism, and elimination of PCB-153 in a lactating woman, it is also useful in its capacity to provide an estimate of intake dose given a certain tissue (or in this case, milk) level of PCB-153. From our model simulation, the PCB level found in the milk of the Canadian Inuits suggests an intake dose almost 50 times higher than the median value of Akutsu et al. (2005); that is, 0.294 μg/kg/hr versus 0.0068 μg/kg/hr. This is probably a reflection of a high rate of consumption of fish in the Inuit diet. Similar estimations can be made if other parameters are known for a certain population or individual. Additionally, the model can be expanded to include an infant, which simulates absorption, distribution, metabolism, and elimination in a nursing child, such as the approach discussed by Clewell and Gearhart (2002). Finally, because this model calculated the partition coefficients for PCB-153 based on structural properties of the chemical, the model can be expanded to other PCBs. Using the formulas described by Parham et al. (1997), partition coefficients can be calculated for any PCB.

## Figures and Tables

**Table 1:** Parameters used for lactating mothers.

Parameter	Value	Source/reference
Physiologic values		
BW (kg)	63.9 (adjusted)^a^	Taizhong hospital, present study
BH (cm)	167	Taizhong hospital, present study
Cardiac output fraction	18.0	Byczkowski and Fisher 1995
Blood volume	(35.5 × BH + 2.278 × BW – 3,382) × 0.001/0.6178	Price et al. 2003
Tissue volume (% of BW)		
VLC	0.04	Byczkowski and Fisher 1995
VFC	0.22	Byczkowski and Fisher 1995
VMtC	0.025	Gentry et al. 2003
VRC	0.91^b^ – (VLC + VFC + VMtC)	
Blood flow (% of cardiac output)		
QLC	0.25	Byczkowski and Fisher 1995
QFC	0.1	Fisher et al. 1997
QMtC	0.07	Fisher et al. 1997
QRC	1 – (QLC + QFC + QMtC)	
Milk volume (L)	Adjusted^c^	Kent et al. 1999
Milk production rate (L/hr)	0.0328	Kent et al. 2006
PCB-153 metabolic rate (L/hr)	0.000164	Extrapolated from mouse value
Partition coefficients		
Fat	303	Calculated from Parham et al. 1997
Mammary tissue	302	Calculated from Parham et al. 1997
Liver	17.9	Calculated from Parham et al. 1997
Body (average of partition coefficients for brain, muscle, and skin)	16.3	Calculated from Parham et al. 1997

Abbreviations: BH, body height; BW, body weight; QFC, fat blood flow; QLC, liver blood flow; QMtC, mammary tissue blood flow; QRC, body blood flow; VFC, fat tissue volume; VLC, liver tissue volume; VMtC, mammary tissue volume; VRC, body tissue volume.

a Adjusted according to the average of 20 subjects in a Taiwanese hospital.

b The value of 0.91 was used instead of 1 to take into account parts of the body not included in the model, such as skeleton and hair.

c Milk volume is a time-dependent function based on data from Kent et al. (1999). Details of the model code are available from the corresponding author on request.

**Table 2:** Parameters used for females 0–25 years of age.

Parameter	Value	Reference
Body weight (kg)		
0–1 year	9.8	Haddad et al. (2001)
1–5 years	18.8	Haddad et al. (2001)
5–10 years	31.9	Haddad et al. (2001)
10–15 years	51.5	Haddad et al. (2001)
> 15 years	54.4	Haddad et al. (2001)
Blood volume (L)		
0–1 year	0.3	Haddad et al. (2001)
1–5 years	1.33	Haddad et al. (2001)
5–10 years	2.49	Haddad et al. (2001)
10–15 years	3.0	Present study
> 15 years	4.2	Haddad et al. (2001)
Cardiac output (L/hr)		
0–1 year	84	Price et al. (2003)
1–5 years	318.6	Price et al. (2003)
5–12 years	310.8	Price et al. (2003)
12–21 years	385.2	Price et al. (2003)
> 21 years	439.8	Price et al. (2003)
Fat volume fraction^a^		
0–1 year	0.22	Haddad et al. (2001)
1–5 years	0.157	Haddad et al. (2001)
5–10 years	0.198	Haddad et al. (2001)
10–25 years	0.33	Haddad et al. (2001)
Mammary tissue volume		
0–13 years	0.0001	Present study
> 13 years	0.02	Gentry et al. (2003)

All other parameters and partition coefficients are the same as those listed in Table 1.

a Obtained by dividing adipose tissue weight by age-appropriate body weight.
